# Impact of task-oriented training based on sodium hyaluronate therapy on kinesiophobia in patients after anterior cruciate ligament reconstruction

**DOI:** 10.12669/pjms.42.3.12621

**Published:** 2026-03

**Authors:** Yinghui Wang, Weihua Song, Hui Li, Chunhong Zhao

**Affiliations:** 1 Yinghui Wang, Luohe Orthopedic Hospital, Luohe, China; 2 Weihua Song Xuanwu Hospital Capital Medical University, Beijing, China; 3 Hui Li, Luohe Orthopedic Hospital, Luohe, China; 4 Chunhong Zhao, Luohe Orthopedic Hospital, Luohe, China

**Keywords:** Anterior cruciate ligament reconstruction, Kinesiophobia, Sodium hyaluronate, Task-oriented training

## Abstract

**Objective:**

To investigate the effect of task-oriented training (TOT) based on sodium hyaluronate therapy on kinesiophobia in patients after anterior cruciate ligament reconstruction (ACLR).

**Methodology:**

A total of 110 patients who underwent ACLR between January 2022 and January 2024 at Luohe Orthopedic Hospital, Luohe, China, were randomly divided into a control group (n=55) and an observation group (n=55) using a random number table. The study was a prospective, randomized controlled trial conducted from January 2022 to January 2024. All patients received sodium hyaluronate therapy. The control group received routine nursing care, while the observation group underwent TOT. The primary outcome measures included kinesiophobia scores (at rest and during activity), knee function scores (HSS scores) and pain scores (NRS scores) at one, three and six months postoperatively. Kinesiophobia scores (at rest and during activity) and knee function scores (HSS scores) at one, three and six months postoperatively were compared between the two groups. SPSS 22.0 with repeated measures ANOVA.

**Results:**

The observation group had significantly lower kinesiophobia scores and pain scores (at rest and during activity) than the control group (P<0.05). Additionally, the HSS scores in the observation group were significantly higher than those in the control group at all follow-up time points (P<0.05).

**Conclusion:**

TOT combined with sodium hyaluronate therapy can reduce the incidence of kinesiophobia and functional impairment after ACLR, promote knee function recovery and improve patients’ quality of life.

## INTRODUCTION

Anterior cruciate ligament reconstruction (ACLR) aims to restore patients’ ability to engage in sports. However, after ACLR, issues such as limited range of motion, pain and muscle weakness may occur.^1^-^6^ Early postoperative physical therapy focused on functional improvement is necessary. Psychological factors also significantly influence return to sport (RTS) after ACLR.^7^-^8^ Studies have shown that the incidence of kinesiophobia after ACLR is as high as ≥56%, which not only seriously affects patients’ functional exercise and quality of life but can even lead to disuse syndrome. Currently, numerous intervention studies on postoperative kinesiophobia in ACLR patients have been conducted abroad, while related research in China is scarce and primarily focuses on the impact of rehabilitation outcomes in ACLR patients. Research on kinesiophobia after anterior cruciate ligament reconstruction is even rarer and orthopedic nurses-as the main implementers of interventions-overemphasize analgesic effects.^9^ Task-oriented training, a training model based on motor control and learning, can shorten postoperative hospital stays, reduce medical costs and achieve rapid rehabilitation.^10^ This study aimed to investigate the impact of task-oriented training based on sodium hyaluronate therapy on kinesiophobia in patients after anterior cruciate ligament reconstruction, providing a reference for the scientific and standardized management of kinesiophobia in ACLR patients.

## METHODOLOGY

A total of 110 patients who underwent ACLR between January 2022 to January 2024 at Luohe Orthopedic Hospital, Luohe, China, were randomly divided into a control group (n=55) and an observation group (n=55) using a random number table. The study was a prospective, randomized controlled trial. All patients were followed up for at least six months.

### Inclusion criteria:

- Age ≥19 years; diagnosed with kinesiophobia (Tampa Scale of Kinesiophobia-II [TSK-II]); voluntary informed consent.

### Exclusion criteria:

- Combined organ injury; severe mental disorders or paraplegia; incomplete clinical data; dropout during the study.

The control group included 24 males and 31 females, aged 23-81 years (mean: 51.28±3.12 years), with 25 left-sided and 30 right-sided surgeries. The observation group included 26 males and 29 females, aged 24-81 years (mean: 52.43±3.29 years), with 23 left-sided and 32 right-sided surgeries. There were no significant differences in gender, age, or affected limb between groups (P>0.05). The study was approved by the hospital ethics committee (No. 2022-13) and all patients provided written informed consent.

### Ethical Approval:

It was approved by the medical research (New Technology) Ethics Committee of the Second Affiliated Hospital of Luohe medical college. The ethics approval number is 2022-13, and the approval date is January 14, 2022.

### Interventions:

All patients received intra-articular injections of sodium hyaluronate (Shandong Bausch & Lomb Freda Pharmaceutical, China; 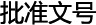 H20067379) at a dose of 2.5 mL (25 mg) administered once weekly. Sodium hyaluronate injections were initiated after ACLR when ultrasound examination showed intra-articular effusion ≤4 mm and the knee joint exhibited no redness, swelling, heat or pain.

### Control group:

Routine nursing care (including basic rehabilitation guidance).

### Observation group:

TOT added to routine care, consisting of:

- Personalized exercise plan: Customized functional training based on surgical type and physical condition, including chest expansion and limb flexion/extension exercises (ten minutes/session, three sessions/day).
- Postoperative days one-three: Bed-to-standing transition with crutch walking (20-30 minutes/session, three sessions/day).
- Postoperative days four-five: Muscle relaxation training with static stretching and soothing music (20-30 minutes/session, three sessions/day).
- Postoperative days six-twelve: Crutch-assisted stair training (three sessions/day).
- Patients are required to push objects such as building blocks and plastic cups in four directions with the affected limb to reach preset target points.
- Object relocation: Patients move items on the table from one place to another.

Personalized lower limb training is further conducted according to the activities and tasks the patients wish to perform. Corresponding tasks are designed by analyzing the patients’ task-execution capabilities to help them adapt to daily life and the surrounding environment. During task-oriented training for the affected limb, the weight and distance of pushed, grabbed, or rotated objects are gradually increased. Patients are guided to practice daily living skills such as brushing teeth, washing faces, eating and using the toilet. Family members must accompany patients throughout functional exercises to prevent secondary fractures.^11^-^13^ Task-oriented training is conducted five sessions per week for four weeks. Follow-ups are performed at one month, three months and six months after discharge.

### Evaluation tools:

- **TSK-II score:** Assessed kinesiophobia (17 items, total score 68; score >37 indicated kinesiophobia). The Tampa Scale for Kinesiophobia (TSK) was originally developed by Miller et al. as a 17-item self-report questionnaire using a 4-point Likert scale (1-4, from strongly disagree to strongly agree) and has been widely applied to assess kinesiophobia, particularly in patients with low back pain.^14^ A total score >37 is generally accepted as the cutoff value for defining the presence of kinesiophobia, and higher scores indicate more severe fear of movement. In 2015, Wei et al. translated and culturally adapted the TSK into Chinese, and the Chinese version demonstrated good psychometric properties, with a Cronbach’s α coefficient of 0.778 and test–retest reliability of 0.860, indicating good reliability and validity in Chinese populations.^15^
- **HSS knee score:** Evaluated knee function (100-point scale: ≥85 = excellent, 70-84 = good, 60-69 = fair, <60 = poor).^16^-^18^
- **Numerical Rating Scale (NRS):** Measured pain intensity at rest and during activity (0 = no pain, 10 = worst pain).^18^

### Statistical analysis:

Data were analyzed using SPSS 22.0. Continuous variables were expressed as mean ± standard deviation *(χ̅±S)* and compared using repeated measures ANOVA. A P<0.05 was considered statistically significant.

## RESULTS

### Kinesiophobia and pain scores:

After intervention, the observation group had significantly lower TSK-II scores and NRS scores (at rest and during activity) than the control group (all P<0.05, Table-I).

**Table-I:** Comparison of kinesiophobia and pain scores between groups (*χ̅*±*S*, points).^1^

Group	Pre-intervention			Post-intervention		
	TSK-II Score	Resting NRS	Active NRS	TSK-II Score	Resting NRS	Active NRS
Control	34.22±4.31	5.38±1.22	7.22±1.31	25.18±1.79	2.18±0.59	4.31±1.01
Observation	35.19±4.47	5.17±0.82	7.19±1.29	21.53±1.38	1.53±0.38	3.28±0.97
t-value	1.116	1.041	0.117	11.533	6.614	5.253
P-value	0.267	0.301	0.908	<0.001	<0.001	<0.001

1 Post-intervention time point: Nurses shall assess the changes in kinesiophobia scores of patients in the observation group after implementing task-oriented training.

### Knee function scores:

The observation group showed significantly higher HSS scores than the control group at one, three and six months postoperatively (all P<0.05, Table-II).

**Table-II:** Comparison of HSS scores between groups (*χ̅*±*S*, points).

Group	Preoperative	1 Month Postop	3 Months Postop	6 Months Postop
Control (n=55)	44.15±5.27	62.32±7.28	65.13±6.59	68.31±2.39
Observation (n=55)	43.98±5.13	65.21±5.94	69.55±4.67	73.75±4.44
*t*-value	0.165	2.383	4.239	8.357
*P*-value	0.869	0.019	<0.001	<0.001

## DISCUSSION

### Impact on Kinesiophobia Scores and Pain Scores:

The observation group exhibited significantly reduced kinesiophobia and pain compared to the control group. This may be attributed to the structured TOT protocol, which progressively guided patients from static stretching to resistive training and functional walking, enhancing exercise compliance and promoting early knee function recovery.^19^-^22^ Our findings are consistent with those reported by Kazarian et al., who demonstrated that addressing psychological factors significantly improves functional outcomes after joint surgery.^22^ Similarly, Marques-Sule et al. found that structured rehabilitation programs effectively reduce kinesiophobia in cardiac patients, suggesting that task-oriented approaches have broad applicability across different patient populations.^21^ However, some international studies have reported less pronounced improvements in kinesiophobia. For instance, Jochimsen et al. observed that patients with femoroacetabular impingement syndrome showed persistent kinesiophobia despite rehabilitation interventions, possibly due to differences in patient engagement, therapy intensity, or underlying pathology.^23^ The discrepancy between our results and these studies may be explained by several factors: first, our protocol specifically combined physical rehabilitation with viscosupplementation therapy, which may provide superior pain relief and joint lubrication, thereby enhancing patients ‘confidence in movement; second, the cultural context in China, where family members are actively involved in rehabilitation as observed in our study, may provide additional psychological support that facilitates recovery. Importantly, by showing that TOT paired with sodium hyaluronate effectively eases kinesiophobia—an emotional barrier commonly neglected in orthopedic recovery—this study contributes clinically valuable evidence supporting the necessity of integrated rehabilitation approaches rather than concentrating only on physical improvement. The clinical significance lies in the strong connection between kinesiophobia and delayed progress, poorer functional performance, and extended disability. By demonstrating that TOT directly addresses this challenge, our study emphasizes a practical method that clinicians can adopt to enhance postoperative development.

### Impact on Postoperative Knee Joint Function:

The HSS scores of the observation group were significantly higher than those of the control group at one month, three months and six months post - operation, with statistically significant differences ($P < 0.05$). This indicates that task - oriented training based on sodium hyaluronate therapy is more conducive to the management of kinesiophobia, improving the quality of life of patients and their rehabilitation outcomes. This finding aligns with the work of Lei et al., who reported that early ambulation protocols following total knee arthroplasty significantly improved functional recovery in Chinese patients.^24^ Similarly, Li et al. demonstrated that enhanced recovery nursing combined with limb training improved knee joint function and neurological outcomes after total knee arthroplasty.^19^ International studies by Wu et al. have also emphasized the importance of comprehensive rehabilitation principles in ACL repair, supporting our integrated approach.^1^ However, Birchmeier et al. noted that quadriceps muscle recovery following ACL injury and reconstruction varies considerably across studies, with some patients experiencing persistent weakness despite rehabilitation interventions.^6^ This difference may be attributed to variations in surgical techniques, graft types, and rehabilitation intensity across different institutions. Additionally, Achermann et al. from Switzerland found that return-to-sport decisions after ACLR are influenced by multiple factors beyond physical recovery, including psychological readiness and social support, which our task-oriented training protocol specifically addresses.^13^ The reason may be that early identification after ACLR, along with the adoption of active, targeted and individualized measures, can reduce the incidence of kinesiophobia in THA patients. Studies by Sertel et al. have demonstrated that in older adults with chronic pain, there is a significant relationship between physical activity levels and kinesiophobia, further supporting the rationale for activity-based interventions.^12^ Furthermore, studies from regions with different healthcare systems, such as those conducted by Strozyk et al. in Germany, have suggested that the effectiveness of postoperative interventions may vary based on the availability of rehabilitation resources and patients ‘socioeconomic backgrounds.^25^ This is beneficial for conducting rehabilitation training actively, scientifically and standardly, thereby improving the quality of rehabilitation.

Our study therefore adds new evidence supporting the role of task-oriented rehabilitation in improving postoperative knee function when combined with joint-lubricating biological therapy. This combined strategy may better address the multidimensional recovery needs of ACLR patients, offering a more holistic model for postoperative care.

### Clinical Implications:

The utilization of the TSK-II scale provides a standardized and scientific approach for the dynamic assessment of kinesiophobia in clinical settings, offering a solid basis for tailored intervention strategies. Our results also show that the combination of task-oriented training with sodium hyaluronate therapy not only improves the flexion and extension range of motion in the knee but also promotes a more complete and efficient rehabilitation process. Regional evidence, including the predictive model developed by Xie et al., indicates that early identification of high-risk patients—particularly those with high pain scores, low HSS/ADL scores, or anxiety—can greatly improve the effectiveness of targeted interventions.^26^ Baysalhan Öztürk et al. similarly demonstrated that kinesiophobia in rheumatoid arthritis patients is closely related to quadriceps muscle strength, fear of falling, and disease activity, reinforcing the importance of comprehensive assessment and intervention strategies.^27^ The integration of task-oriented training with sodium hyaluronate therapy following ACLR not only improves the flexion and extension range of motion in the knee but also enhances overall joint function, promoting a more complete and efficient rehabilitation process. International consensus statements, such as the Panther Symposium ACL Injury Return to Sport Consensus Group recommendations reported by Meredith et al., have emphasized the need for multifactorial assessment including psychological readiness before return to sport.^2^ However, variations in post-surgical recovery protocols across countries and settings can result in differing rates of functional recovery, highlighting the need for region-specific adaptations of rehabilitation programs. Clinically, this suggests that rehabilitation protocols should extend beyond traditional strength and mobility training to incorporate psychologically informed practices that directly address fear-avoidance behaviors. Given the high global incidence of ACL injury and the substantial socioeconomic burden of prolonged rehabilitation, our findings have strong applicability for optimizing recovery strategies and improving long-term outcomes.

### Novel Contributions to Medical Literature:

This study makes several important contributions to the existing body of knowledge. First, to our knowledge, it is one of the first studies to examine the combined effects of task-oriented training and sodium hyaluronate therapy specifically in patients with anterior cruciate ligament reconstruction (ACLR) and movement phobia in a Chinese population. While previous studies have examined these interventions separately, this study demonstrates the synergistic effects of combining them. Second, the findings bridge the gap between international rehabilitation practice and the Chinese clinical setting, providing evidence for the adaptation of task-oriented training protocols for orthopaedic rehabilitation in China. Third, the study extends the applicability of the TSK-II scale for dynamic monitoring of kinesiophobia throughout the postoperative period, thereby establishing an evidence-based framework for psychological assessment in ACLR rehabilitation.

### Strengths of the study:

The randomized controlled trial using a large sample size (n=110) adds strength to the internal validity of the study. The 6 month follow up is capable of assessing both short term and medium term outcomes. Validated outcome measures, such as the TSK-II, HSS and the NRS provide reliable and reproducible scores. A thorough description of the Task Oriented Training (TOT) protocol facilitates reproduction of the protocol in clinical settings as well as in future research. Integrating both physical and psychological outcome measures gives an overall picture of patient’s recovery.

### Limitations:

This study is carried out only at one center in a Chinese hospital; thus, its generalization capability to the entire population or different healthcare institutions is still insufficient. Furthermore, because the clinical trial did not apply blinding, it could introduce performance bias into the trial’s results. Additionally, apart from six months, no other follow-up is available. It is unclear whether the benefits observed can maintain their effects over a longer period of time. Fourthly, due to restrictions on manpower, resources, finances, and time, no subgroup analyses were carried out based on age, gender, or graft type.

## CONCLUSION

The use of the TSK score scale for kinesiophobia has made the dynamic assessment of patients more standardized and scientific, providing a reliable basis for clinical intervention measures. The application of task - oriented training based on sodium hyaluronate therapy after anterior cruciate ligament reconstruction can enhance the flexion and extension range of the knee joint as well as the overall function of the knee joint.

### Author’s Contribution:

**YW:** Conceived, designed, statistical analysis & editing of manuscript, is responsible for integrity of research.

**WS and HL:** Did data collection, Critical Review and manuscript writing.

**CZ:** Literature search**,** Critical review.

All authors have read and approved the final version of the manuscript.
